# Supplementary material for: The interactome of intact mitochondria by cross-linking mass spectrometry provides evidence for coexisting respiratory supercomplexes
Source: Mol Cell Proteomics. 2017 Dec 8;17(2):216–32. doi: 10.1074/mcp.RA117.000470 (PMC5795388; doi:10.1074/mcp.RA117.000470)
Supplement: Supplemental Data [file supp_RA117.000470_133922_0_supp_23978_fzffwf.zip › spectra_annotation/mito_DR_spectra_annotation/369-1-2-1-11-1.pdf]

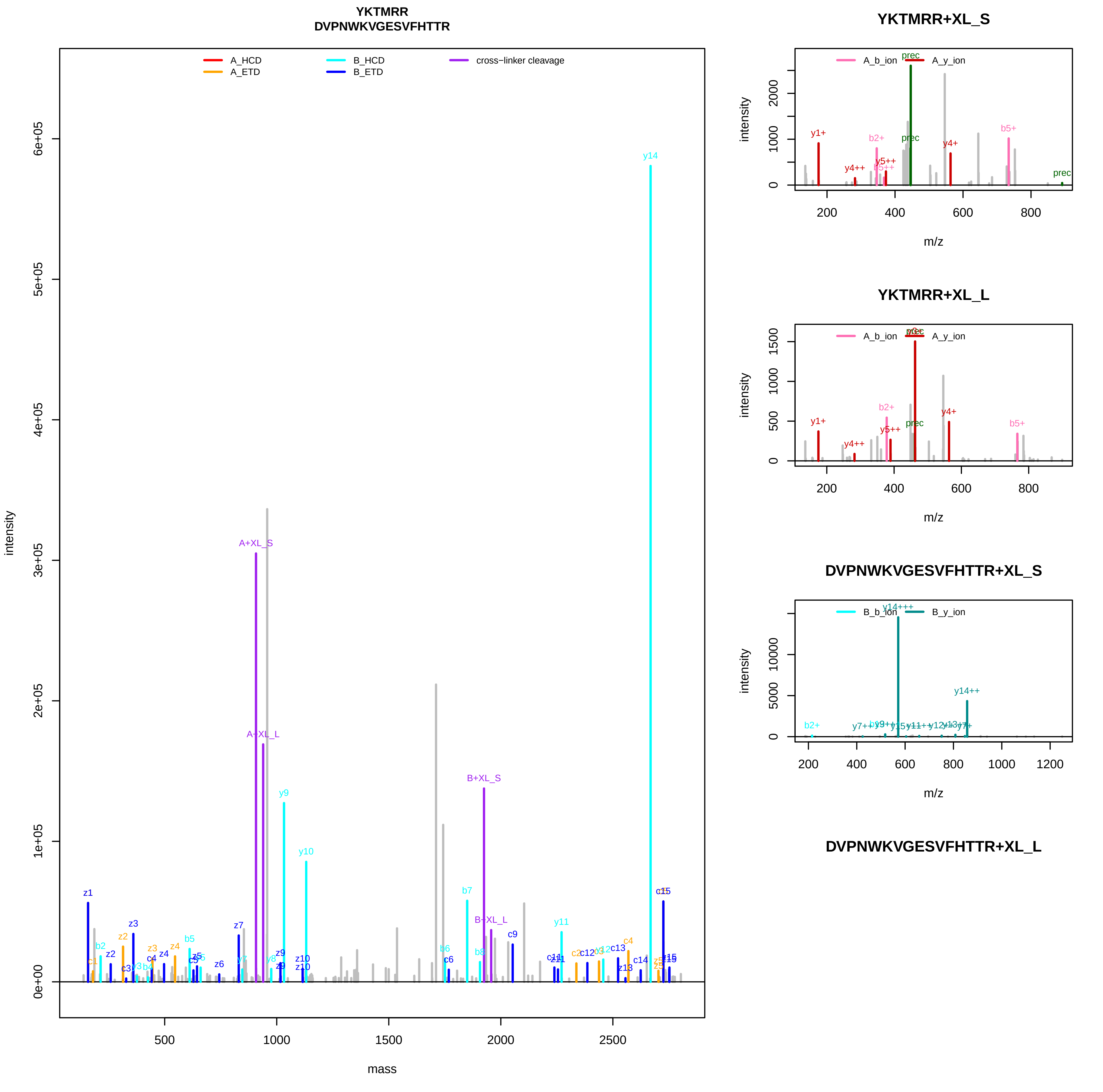
YKTMRR
DVPNWKVGESVFHTTR
A_HCD
A_ETD
B_HCD
B_ETD
cross−linker cleavage
intensity
mass
0e+00
1e+05
2e+05
3e+05
4e+05
5e+05
6e+05
500
1000
1500
2000
2500
A+XL_S
A+XL_L
B+XL_S
B+XL_L
y14
y9
y10
y11
b7
b5
b2
c9
z1
z2
z3
z4
z6
z7
z9
z10
c4
c5
c6
c11
c12
c13
c14
c15
z13
z15
YKTMRR+XL_S
A_b_ion
A_y_ion
intensity
m/z
y1+
y4++
y4+
b2+
b5+
b5++
y5++
prec
YKTMRR+XL_L
DVPNWKVGESVFHTTR+XL_S
B_b_ion
B_y_ion
y14+++
y14++
b2+
DVPNWKVGESVFHTTR+XL_L
